# MicroRNAs in skeletal muscle and their regulation with exercise, ageing, and disease

**DOI:** 10.3389/fphys.2013.00266

**Published:** 2013-09-30

**Authors:** Evelyn Zacharewicz, Séverine Lamon, Aaron P. Russell

**Affiliations:** Centre for Physical Activity and Nutrition Research, School of Exercise and Nutrition Sciences, Deakin UniversityBurwood, VIC, Australia

**Keywords:** miRNA, skeletal muscle, exercise, disease, ageing

## Abstract

Skeletal muscle makes up approximately 40% of the total body mass, providing structural support and enabling the body to maintain posture, to control motor movements and to store energy. It therefore plays a vital role in whole body metabolism. Skeletal muscle displays remarkable plasticity and is able to alter its size, structure and function in response to various stimuli; an essential quality for healthy living across the lifespan. Exercise is an important stimulator of extracellular and intracellular stress signals that promote positive adaptations in skeletal muscle. These adaptations are controlled by changes in gene transcription and protein translation, with many of these molecules identified as potential therapeutic targets to pharmacologically improve muscle quality in patient groups too ill to exercise. MicroRNAs (miRNAs) are recently identified regulators of numerous gene networks and pathways and mainly exert their effect by binding to their target messenger RNAs (mRNAs), resulting in mRNA degradation or preventing protein translation. The role of exercise as a regulatory stimulus of skeletal muscle miRNAs is now starting to be investigated. This review highlights our current understanding of the regulation of skeletal muscle miRNAs with exercise and disease as well as how they may control skeletal muscle health.

## Introduction

Maintaining skeletal muscle metabolism, size and contractile function are prerequisites for whole body health throughout life. Skeletal muscle is highly sensitive to extracellular and intracellular signals elicited by contractions from endurance and resistance exercise. These signals are the catalyst for numerous physiological adaptations including enhanced substrate metabolism, mitochondrial biogenesis, angiogenesis, muscle growth and regeneration (Hawke, 2005; Hawley et al., 2006; Léger et al., 2006; Russell, 2010). In contrast, a lack of exercise and muscle contraction, as seen in numerous neuromuscular, musculoskeletal and chronic diseases, as well as in limb immobilization following trauma, sedentary lifestyles or with ageing, negatively impacts on skeletal muscle metabolism, size and contractile function. These negative responses play a role in the on-set and progression of secondary diseases, such as diabetes and cardiovascular disease, increase the severity of chronic diseases and limit the availability of treatment options.

Extracellular and intracellular signals, activated by exercise or disease and inactivity, influence transcriptional and translational regulation of genes encoding proteins that control skeletal muscle metabolism, growth, regeneration and contraction (Dela et al., 1994; Russell et al., 2003, 2005; Short et al., 2003; Wadley et al., 2007). The control of these transcription and translation processes is regulated by transcription factor activation (Keller et al., 2001; McGee et al., 2006), histone modification (McGee et al., 2009) and DNA methylation (Nakajima et al., 2010; Barres et al., 2012). However, the discovery of microRNAs (miRNAs) (Lee et al., 1993; Reinhart et al., 2000) has revealed another level of complexity in transcriptional and translational regulation (Bartel, 2004). Our understanding of how exercise and disease regulate miRNA expression and activity as well as their messenger RNA (mRNA) targets implicated in skeletal muscle health is rudimentary. Exploring this field will advance our knowledge of the mechanisms behind skeletal muscle health and disease and potentially reveal novel therapeutic targets that may be used as a means to improve health outcomes for people suffering from muscular disorders.

## The regulation of mRNAs by microRNAs

**Figure 1:**
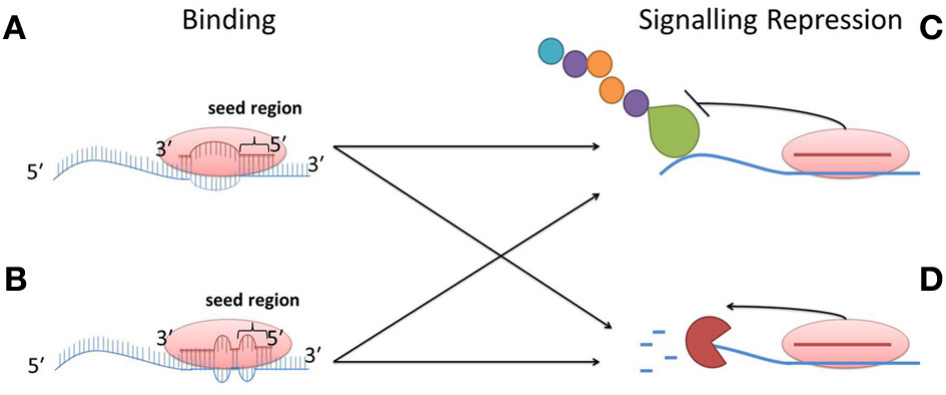
**Schematic representation of possible miRNA—mRNA interactions. (A)** Perfect Watson-Crick complementary binding. **(B)** Imperfect binding, with compensatory binding at the 3′ end of the mRNA molecule. Once bound to the mRNA molecule, the miRNA can either **(C)** inhibit protein translation, or **(D)** induce mRNA degradation.

MiRNAs play an imperative role in the maintenance of healthy cellular function. The primary role of miRNAs is to specifically inhibit protein expression (Olsen and Ambros, 1999; Lee et al., 2004; Wightman et al., 2004; Humphreys et al., 2005; Pillai et al., 2005; Huili et al., 2010) and this can be achieved either by degrading specific mRNA species or by repressing protein translation. Overall, mRNA degradation accounts for the majority of miRNA activity (Huili et al., 2010). The precise mechanism of miRNA targeting and activity still remains to be fully understood. However, miRNA activity appears to be largely dependent on its binding capacity to the target mRNA molecule (Brennecke et al., 2005; Hu and Bruno, 2011). Most miRNA binding sites are located in the 3′ untranslated region (UTR) of the target mRNA species and exist in multiple copies (Hu and Bruno, 2011). There are 2 known binding types for miRNAs (Brennecke et al., 2005). The first is perfect Watson-Crick complementary binding between the 5′ end of the miRNA and the 3′ UTR of the target mRNA; although some miRNA can target the 5′ UTR. This region of the miRNA is called the “seed” region, occurring at base position 2–8 on the 5′ end of the miRNA. This perfect binding within the seed region is sufficient to suppress mRNA activity on its own. The second type of binding is imperfect binding between the seed region and the 3′ UTR but with compensatory binding at the 3′ end of the mRNA molecule. Figure 1 depicts the possible types of miRNA/mRNA interactions along with the effect of miRNA binding on mRNA degradation and translational repression.

Understanding how miRNAs target and bind mRNAs has led to the development of a number of different algorithms and bioinformatics websites such as miRWalk and TargetScan (Lewis et al., 2005; Dweep et al., 2011). These softwares are commonly used to predict specific mRNA/miRNA interactions. However, miRNA binding rules are complex and are not completely understood, resulting in a lack of consensus in the literature. Foremost, establishing direct cause-and-effect links between miRNAs and mRNA targets is key to understanding the underlying molecular mechanisms behind health and disease and thus the development of effective and targeted therapies.

## Regulation of microRNA biogenesis machinery

MiRNA biogenesis is a complex process requiring co-ordination of primary miRNA (pri-miRNA) transcription in the nucleus. The pri-miRNA is cleaved by the RNase-III type endonuclease Drosha associated with Pasha (also known as DGCR8) into a precursor molecule referred to as the pre-miRNA. The pre-miRNA is then exported into the cytoplasm by exportin 5 (XPO5) and cleaved a second time by Dicer (Lee et al., 2003; Lund et al., 2004). This gives rise to a duplex strand that is unwound by RNA helicases. The miRNA strands are separated and incorporated into the RNA-induced silencing complex (RISC), providing specificity for the RISC to identify and bind to the 3′ or 5′ UTR of the target mRNAs (Khvorova et al., 2003; Bartel, 2004; Lee et al., 2009). There has been very little investigation into the exercise-induced regulation of the miRNA biogenesis machinery. Work from our group has shown that in the 3 h following a single bout of endurance exercise in untrained males there is an increase in XPO5 mRNA as well as Drosha and Dicer mRNA (Russell et al., 2013). XPO5 and Drosha mRNA are also increased in old but not young subjects within 6 h following a single bout of resistance exercise (Drummond et al., 2008b). Interestingly, this change occurred in parallel with a decrease in XPO5 protein levels in both groups. In addition to its role in exporting pre-miRNA from the nucleus to the cytoplasm, XPO5 also stabilizes pre-miRNAs (Lund et al., 2004), suggesting that XPO5 increases the pool of pre-miRNAs. The upregulation of XPO5 in skeletal muscle following exercise may be an adaptive response to aid processing of new pre-miRNAs that regulate exercise-induced adaptations in muscle. However, further work is required to elucidate the regulation and expression of miRNA biogenesis machinery in muscle and to understand how this affects the miRNA pool.

## Regulation of microRNAs in healthy muscle and exercise

Many miRNAs can be highly and specifically enriched in certain tissues (Sood et al., 2006). Skeletal muscle enriched miRNAs, referred to as myomiRs, include miR-1, miR-133a, miR-133b, miR-206, miR-208, miR-208b, miR-486 and miR-499 (McCarthy and Esser, 2007; Callis et al., 2008). The transcriptional regulation of muscle enriched miRNAs is under the control of myogenic regulatory factors (MRFs), such as MyoD, myogenin, Myf5 and MRF4 (Rao et al., 2006; Rosenberg et al., 2006), that tightly control skeletal muscle regeneration (Rudnicki and Jaenisch, 1995; Tajbakhsh et al., 1996; Sabourin and Rudnicki, 2000). These miRNAs can be arranged in polycistronic clusters and transcribed together or in parallel with protein-coding genes (Sweetman et al., 2008). MiRNAs expressed in skeletal muscle are modulated during multiple biological processes involved in skeletal muscle growth, development and maintenance, including atrophy and hypertrophy (McCarthy and Esser, 2007; McCarthy et al., 2007, 2009).

Skeletal muscle atrophy and hypertrophy models have been used to characterize the role and regulation of miRNAs potentially involved in maintaining skeletal muscle mass (see Table 1). Following 7-days of hypertrophy-inducing functional overload of the mouse plantaris muscle, expression of miR-1 and miR-133a was decreased by 50% when compared to control muscle (McCarthy and Esser, 2007). MiR-1 and miR-133a were proposed to contribute to muscle hypertrophy by the removal of their transcriptional inhibitory effect on growth factors such as IGF-1. In support of this hypothesis a regulatory feedback loop was demonstrated *in vitro* where IGF-1 downregulated miR-1 via the Akt/FoxO3a pathway (Elia et al., 2009). It was also shown that FoxO3a increased levels of miR-1 resulting in reduced IGF-1 protein levels.

**Table 1:**
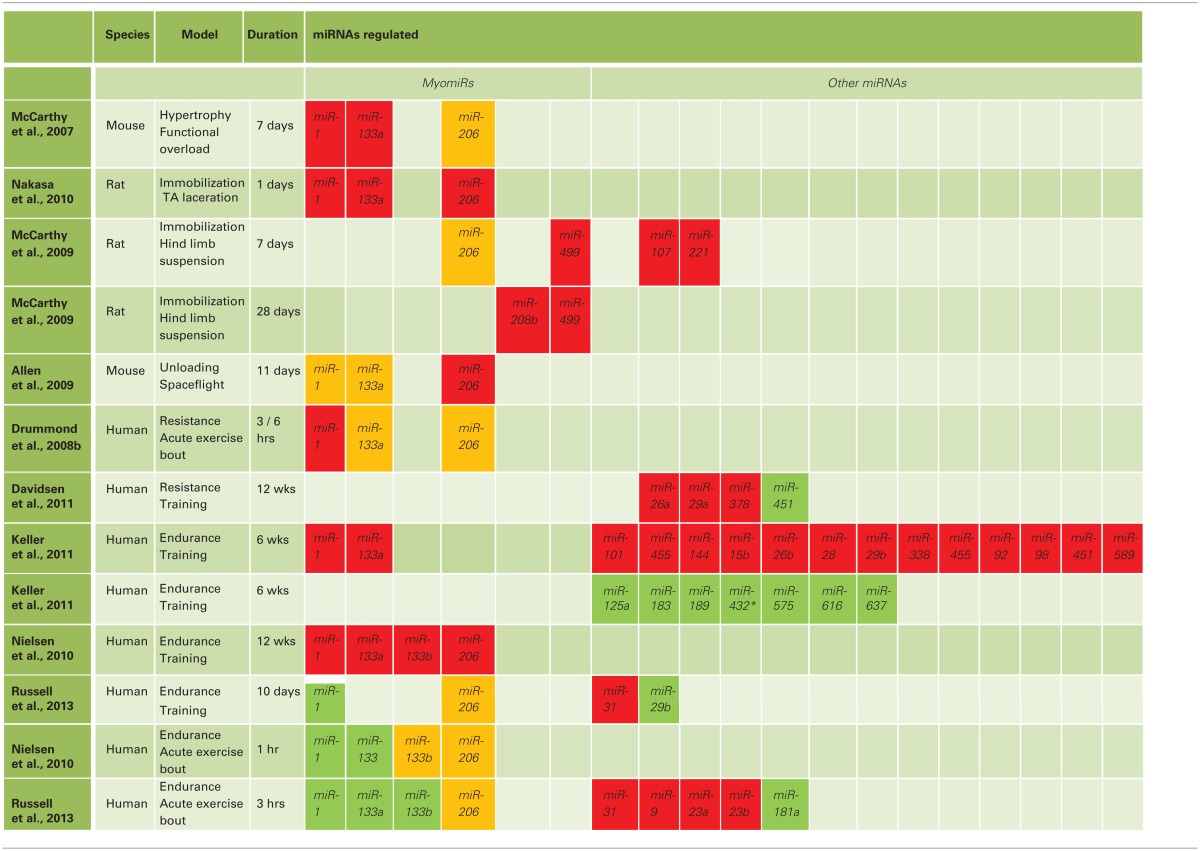
**Regulation of miRNAs by exercise and disuse**.

Red, downregulated; Green, upregulated; Orange, no change.

Unloading of skeletal muscle by immobilization, hind limb suspension (HS) or exposure to microgravity during space flights decreases muscle mass (Allen et al., 2009; McCarthy et al., 2009). Muscle immobilization in rats, induced by the laceration of the tibialis anterior, is associated with a decrease in miR-1, miR-133a and miR-206 levels 1 day post-intervention (Nakasa et al., 2010). MiR-107, miR-221, miR-499 and miR-208b were all downregulated following 7 days of rat HS (McCarthy et al., 2009). Eleven days of spaceflight decreased miR-206 expression (Allen et al., 2009). This decrease was paralleled by an upregulation of FoxO1, atrogin-1 and myostatin mRNAs; all regulators of muscle atrophy (Bodine et al., 2001; Kim et al., 2012). MiR-206 promotes differentiation of C2C12 myoblasts (Kim et al., 2006) and skeletal muscle regeneration following injury in mice (Liu et al., 2012). Whether miR-206 plays a direct or indirect role in repressing the atrophy genes is unknown. However, atrogin-1 degrades MyoD (Tintignac et al., 2005), which in turn positively regulates miR-206 (Chen et al., 2006); however, the existence of a miR-206/MyoD/atrogin-1 regulatory loop has not been investigated.

Exercise plays an important role in maintaining muscle health throughout the lifespan, with resistance exercise a potent anabolic stimulus enhancing muscle protein synthesis and muscle growth (Fry, 2004; Léger et al., 2006; Kumar et al., 2009; Phillips, 2009; Koopman et al., 2011). Few studies have investigated the changes in skeletal muscle miRNA species following resistance exercise in humans. MiR-1 expression is reduced 3 and 6 h following an single bout of resistance exercise, while no changes were observed in miR-133a and miR-206 levels (Drummond et al., 2008b). Following a 12-week resistance-training program aimed at inducing muscle hypertrophy, a difference in miRNA regulation was observed in skeletal muscle of subjects defined as “high responders” vs. “low responders” to the resistance exercise training; “low responders” having little or no muscle hypertrophy following the training intervention (Davidsen et al., 2011). The training protocol resulted in an increase in skeletal muscle miR-451 expression and a decrease in miR-26a, miR-29a and miR-378 expression in the “low responder” group only. Low muscle hypertrophy response to resistance exercise training in healthy young subjects is referred to as anabolic resistance (Baar and Esser, 1999; Terzis et al., 2008); a phenomenon also linked to age-related muscle wasting or sarcopenia in the elderly. Whether miR-451, miR-26a, miR-29a and miR-378 contributes to an attenuated hypertrophy response in young healthy subjects and the mechanisms they control now requires experimental validation.

Endurance exercise is another modulator of skeletal muscle miRNA expression. Following 12 weeks of endurance training, expression of the myomiRs miR-1, miR-133a, miR-133b and miR-206 were all significantly down regulated. These miRNAs returned to pre-training baseline levels 2 weeks after the cessation of training (Nielsen et al., 2010). In contrast, 10 days of endurance training increased miR-1, concomitantly with an increase in miR-29b and a decrease in miR-31 (Russell et al., 2013). With respect to a single bout of endurance exercise, miR-1 and miR-133a levels increased in the untrained state, however this acute response was not observed in the trained state (Nielsen et al., 2010). In addition, we observed that in the 3 h period following a single bout of endurance exercise, miR-1, -133a, -133-b and miR-181a were all increased. In contrast miR-9, -23a, -23b and -31 were decreased (Russell et al., 2013). We also demonstrated *in vitro*, via a reporter assay, that miR-31 directly interacts with HDAC4 (Russell et al., 2013), a component of the MAPK pathway (Symons et al., 2009), as well as with NRF1, which is involved in mitochondrial biogenesis and metabolism. These studies demonstrate that myomiR expression is sensitive to acute and chronic endurance exercise, as well as inactivity. However, their precise targets and the molecular processes regulated remain to be established. Other studies found no correlation between miRNA expression and components of the signaling pathways involved in skeletal muscle adaptation to endurance exercise, such as the MAPK pathway (Kramer and Goodyear, 2007) or the TGF-β pathway (Schabort et al., 2009), suggesting that the individual myomiRs may not regulate these targets in response to endurance exercise. However, multiple miRNAs may need to work together to regulate several key proteins involved in pathway signaling. Following 6 weeks of supervised endurance training in young sedentary males ~800 gene transcripts were regulated and referred to as the training-responsive transcriptome (TRT) (Timmons et al., 2010). Three DNA sequences identified as runt-related transcription factor 1 (RUNX1), sex determining region Y box-9 (SOX9), and paired box gene-3 (PAX3) transcription factor binding sites were overexpressed in the TRT post-training and bioinformatics analyses confirmed RUNX1, SOX9, and PAX3 as potential modulators of muscle aerobic adaptation. MiRNA screening of these subjects also identified 14 miRNAs that were decreased and 7 that were increased in skeletal muscle (Keller et al., 2011). Of the 14 miRNAs that were decreased miR-92, -98, -101 and 104 were predicted to target RUNX1, SOX9 and PAX3. This suggests that the down regulation of these 4 miRNAs during endurance training may permit aerobic adaptation to occur.

## Regulation of microRNAs in myopathies

MiRNAs are essential regulators of skeletal muscle health and their implication in the onset and progression of myopathies and chronic diseases associated with muscle wasting and dysfunction is of high interest. Eisenberg and colleagues observed 185 miRNAs to be commonly dysregulated across ten human primary muscular disorders (Eisenberg et al., 2007). Of these, miR-146b, miR-155, miR-214, miR-221 and miR-222 were consistently increased in almost all of the disease conditions and samples tested. Myotonic dystrophy type 1 (DM1) is the most frequently inherited neuromuscular disorder in adults. MiR-206, a regulator of muscle regeneration (Liu et al., 2012), was specifically augmented in DM1 patients when compared to healthy controls (Gambardella et al., 2010). Additionally miR-1 and miR-335 are upregulated and miR-29b, miR-29c and miR-33 downregulated in DM1 patients, when compared to control subjects suspected of a neuromuscular disorder but not presenting any pathological features (Perbellini et al., 2011). Furthermore, the cellular localization of miR-1, miR-133b and miR-206 appears disrupted in DM1 muscle. Similarly, 11 miRNAs, including the muscle enriched miRNA miR-208, are dysregulated in muscle samples from patients with myotonic dystrophy type 2 (DM2) (Greco et al., 2012).

Duchenne muscular dystrophy (DMD) is the most common and severe form of muscular dystrophy characterized by the absence of the structural membrane protein dystrophin. The muscle-enriched miR-206 is downregulated in *mdx* mice, a well-established animal model for DMD (McCarthy et al., 2007; Yuasa et al., 2008) and miR-206 loss-of-function accelerates the dystrophic phenotype (Liu et al., 2012). In addition to miR-206, another 11 miRNAs were found to be dysregulated in both DMD patients and *mdx* mice (Greco et al., 2009). In mice, these dysregulations could be rescued following therapeutic intervention, such as HDAC inhibition or restoration of nitric oxide (NO) signaling; treatments reported to ameliorate the *mdx* phenotype (Colussi et al., 2008). In DMD samples, miR-31 and miR-486 were also identified as regulators of muscle regeneration (Greco et al., 2009). In human DMD myoblasts, miR-31 inhibition increases dystrophin content. MiR-31 modulation is therefore proposed as a possible therapeutic strategy to ameliorate the DMD phenotype (Cacchiarelli et al., 2011). Interestingly, miR-486 expression was not altered in muscle from patients with Becker muscular dystrophy who expresses a partially functional dystrophin protein (Eisenberg et al., 2007). MiR-486 is proposed to play an important regulatory role in the PTEN (phosphatase and tensin homolog deleted on chromosome 10)/Akt pathway in dystrophin deficient (Alexander et al., 2011) and normal muscle (Small et al., 2010). The *sapje* mutant zebrafish is a model presenting a more severe dystrophic phenotype than the *mdx* mouse (Bassett and Currie, 2004). MiR-199a-5p is elevated in both *sapje* zebrafish and human DMD samples when compared to their respective control samples (Alexander et al., 2013). It was demonstrated that miR-199a-5p inhibits the expression of several components of the Wnt signaling pathway, a pathway that regulates satellite cell maintenance and differentiation (Polesskaya et al., 2003; Le Grand et al., 2009).

Chronic diseases associated with muscle wasting are associated with miRNA dysregulation. MiR-1 downregulation is observed in patients with chronic obstructive pulmonary disease (COPD) and is associated with a downregulation of the MRTF-SRF axis (Lewis et al., 2012); an important transcriptional complex regulating muscle gene expression (Cen et al., 2004; Charvet et al., 2006; Miano et al., 2007). Amyotrophic lateral sclerosis (ALS), a severe motor neuron disorder, is characterized by progressive degeneration of upper and lower motor neurons, a decline in strength, severe muscle atrophy, respiratory insufficiency (Pasinelli and Brown, 2006) and mitochondrial dysfunction (Menzies et al., 2002). We have recently identified an increase in miR-23a in skeletal muscle of ALS patients when compared to healthy controls (Russell et al., 2012). It was established *in vitro* that miR-23a negatively regulates peroxisome proliferator-activated receptor gamma coactivator 1-alpha (PGC-1α) (Russell et al., 2012), a key activator of mitochondrial biogenesis and function. Therapeutic inhibition of miR-23a may rescue PGC-1α activity and ameliorate the ALS phenotype, however this remains to be established. Sixteen miRNAs were dysregulated in patients with laminopathies, a class of myopathies presenting mutations in the lamin A/C gene (Sylvius et al., 2011). Of these, miR-100, miR-192 and miR-135, were directly involved in C2C12 myoblast proliferation and differentiation. In muscle from children suffering from dermatomyositis, an upregulation of 33 miRNAs was observed (Eisenberg et al., 2007). However, miR-126 was specifically downregulated in patients in the early stage of the disease when compared to healthy controls (Kim et al., 2012). MiR-126 is proposed to play a specific role in the early but not in the late stage of juvenile dermatomyositis by promoting the expression of the vascular cell adhesion molecule 1 (VCAM-1), a protein normally expressed in developing but not in mature healthy muscle fibers.

MiRNAs are responsible for the regulation of numerous gene networks and pathways in muscle. Consequently, they are important modulators of skeletal muscle health and many miRNAs are dysregulated in specific muscle disease conditions. Some of these miRNAs play a direct role in muscle cell proliferation or differentiation; however, whether the changes observed in miRNA levels actively contribute or are a consequence of the disease development remains mostly unknown. Identifying the miRNAs dysregulated and understanding their role in muscle diseases is therefore a crucial step in the development of targeted therapeutic strategies.

## Age-associated regulation of microRNAs in skeletal muscle

**Figure 2:**
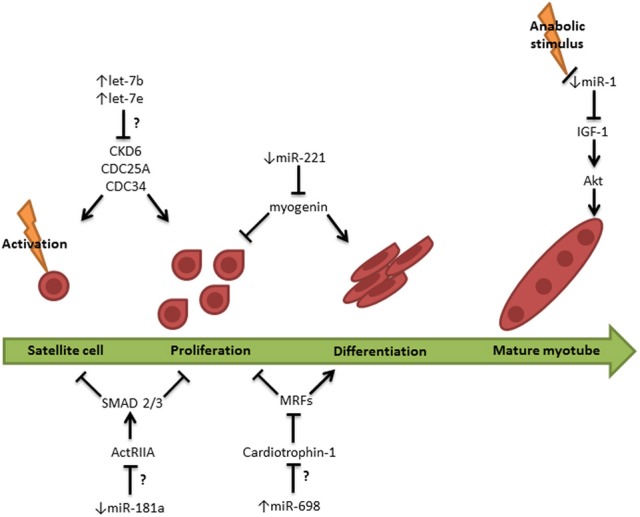
**The potential role of miRNAs in the attenuated myogenic process in the elderly**. Let-7b and let-7e may contribute to the inhibition of satellite cell activation and proliferation via downregulating cell cycle regulators CKD6, CDC25A, and CDC34. In addition miR-181a may inhibit ActRIIA (activin type IIa receptor) and as a consequence permit activation of the proliferation inhibitors SMAD2/3. The combination of miR-221 and miR-698 further inhibits proliferation by down regulating various MRFs and driving terminal differentiation of the myocytes. Anabolic stimulus is known to reduce miR-1. Failure to downregulate miR-1 in elderly skeletal muscle following anabolic stimulation may contribute to anabolic resistance in mature myotubes via inhibition of IGF-1/Akt signaling., ↑ = stimulatory pathway; or, **T** = inhibitory pathway; ↑, upregulated miRNA; ↓, downregulated miRNA; ?, cause-and-effect relationship not established.

Ageing is a condition associated with changes in skeletal muscle size and function as well as the regulation of miRNAs. Ageing studies using *C. elegans* identified changes in miRNAs associated with lifespan and cellular senescence (Boehm and Slack, 2005; Ibáñez-Ventoso et al., 2006; Yamakuchi and Lowenstein, 2009; De Lencastre et al., 2010). Of particular interest, let-7 miRNA is decreased with age in *C. elegans* (Ibáñez-Ventoso et al., 2006; De Lencastre et al., 2010). In contrast, observations in older humans have identified that two miRNAs from the let-7 family of miRNAs, let-7b and let-7e, are elevated in skeletal muscle when compared to young subjects (Drummond et al., 2008a, 2011). The sequence of *C. elegans* (cel)-let-7 varies slightly from human (has)-let-7b and hsa-let-7e and this difference may be sufficient to confer different mRNA targets for these three miRNAs. Caution must be taken when comparing the expression of miRNAs between different species. MiRNAs have historically been named in the same order as they have been discovered. However, occasional discrepancies exist between species; therefore, a direct comparison between correspondingly named miRNAs cannot always be made. In addition, the mRNAs targeted by a same miRNA can differ between species, although their biological function can be conserved. The function of the let-7 family of miRNAs is similar in both *C. elegans* and humans. The primary role of the let-7 miRNAs appears to be anti-proliferative, as observed in human cancer cells (Johnson et al., 2007; Nishino et al., 2008; Dong et al., 2010; Zhao et al., 2010; Lee et al., 2011) and in mouse neuronal stem cells (Nishino et al., 2008). The elevation of let-7 miRNAs may be responsible for the impaired ability to activate and proliferate satellite cells in the elderly skeletal muscle, therefore contributing to the attenuated skeletal muscle regenerative capacity in the elderly (Carlson et al., 2009). Accordingly, bioinformatics analysis identified cell cycle regulation and cellular growth and proliferation as the most highly ranked cellular processes likely to be regulated by the 2 let-7 miRNAs in humans. In old mice with muscle atrophy, 57 miRNAs were differentially regulated in the quadriceps when compared to young mice (Hamrick et al., 2010). Of the dysregulated miRNAs, several were predicted to target genes involved in myogenesis, including Mef2, SRF, cardiotrophin 1, myogenin and the cell cycle regulator type IIA activin receptor. Figure 2 summarizes the known and potential roles of miRNAs in myogenesis in the elderly. In order to elucidate which of these miRNAs are important in the age-related muscle wasting, their mRNA targets and specific roles in muscle regeneration and protein synthesis need to be established.

The most natural way to promote muscle growth is by ingesting good quality protein and engaging in resistance exercise training which is able to stimulate muscle protein synthesis (Yarasheski et al., 1993; Hasten et al., 2000; Drummond et al., 2008a; Katsanos et al., 2008; Kumar et al., 2009; Symons et al., 2009). However, elderly subjects demonstrate an impaired protein synthetic response to resistance exercise (Cuthbertson et al., 2005; Kumar et al., 2009). To date, only one study has investigated the expression of miRNAs in young and old subjects following a protein-stimulating intervention protocol (Drummond et al., 2008b). MiR-1, miR-133a and miR-206 were measured in the muscle of young and old subjects following an acute bout of resistance exercise and ingestion of essential amino acids (EAA). Only miR-1 expression was reduced in the young, but not the old subjects, 3 and 6 h post-exercise and EAA ingestion. A regulatory role for miR-1 as an IGF-1 inhibitor has previously been established (McCarthy and Esser, 2007; Elia et al., 2009). Failure to downregulate miR-1 in elderly subjects following an acute bout of resistance exercise and EAA ingestion may be partially responsible for the attenuated muscle protein synthesis in response to anabolic stimuli. However, more miRNA targets need to be investigated to fully appreciate the role of miRNAs in age-related muscle wasting and to understand the potential mechanisms attenuating protein synthesis following resistance exercise.

## MicroRNAs as biomarkers of disease and exercise-induced adaptation

A key feature of miRNAs is their resistance to ribonucleases (RNases) and therefore their presence and potential stability in blood (Chen et al., 2008; Gilad et al., 2008; Turchinovich et al., 2011). Indeed, miRNAs exist within exosomes, lipoprotein and ribonucleoprotein complexes, which protect them from RNases digestion (Valadi et al., 2007; Zhang et al., 2010; Vickers et al., 2011). MiRNAs can be highly expressed in specific tissues, (Sood et al., 2006) although their role in circulation is not yet clear. Circulating miRNAs can originate from tissues with hematopoietic cells an abundant source of circulating miRNAs (Kosaka et al., 2010; Pritchard et al., 2012).

The existence and stability of miRNAs in circulation has led to the search for miRNA biomarkers for various diseases such as cancer, type 2 diabetes, hepatic diseases and coronary diseases (Chen et al., 2008; Mitchell et al., 2008; D'Alessandra et al., 2010; Huang et al., 2010; Pigati et al., 2010; Brase et al., 2011; Freedman et al., 2012). Aberrant expression of specific miRNAs in the circulation may be reflective of disease burden in a specific tissue. It follows that circulating miRNAs may be beneficial as biomarkers for skeletal muscle disease or skeletal muscle adaptation to exercise. To date, only one study has looked at the circulating miRNAs dysregulated in a muscle disorder. Roberts et al. identified an increase in 57 circulating miRNAs in the *mdx* mouse when compared to wild-type controls (Roberts et al., 2013), including miR-1, miR-133a, and miR-206. The same study demonstrated that miR-1 levels were elevated in the serum of wild-type mice 15 min after a cardiotoxin injection in the tibialis anterior muscle, suggesting that high levels of circulating miR-1 are associated with muscle degeneration and injury.

Changes in plasma and whole blood miRNA profiles have been observed following acute and chronic exercise and in association with training-induced changes in muscle performance (Baggish et al., 2011; Uhlemann et al., 2012; Aoi et al., 2013; Bye et al., 2013; Sawada et al., 2013; Tonevitsky et al., 2013). Table 2 summarizes the circulating miRNAs regulated by exercise, training and fitness level. Circulating miR-133a increases immediately following a marathon race and a single bout of resistance exercise (lateral pulldown, leg press, butterfly) (Uhlemann et al., 2012) but not after a single bout of cycling or treadmill exercise (Baggish et al., 2011; Uhlemann et al., 2012) in trained subjects. In contrast, circulating miR-133a was too lowly expressed at rest and following cycling exercise to be reliably measured (Aoi et al., 2013). Immediately following an acute bout of resistance exercise (bench press and bilateral leg press) in untrained subjects, no changes in circulating miRNA expression was observed (Sawada et al., 2013). Circulating miR-21 and miR-222 are increased following a single bout of cycling exercise and after 90 days of rowing training (Baggish et al., 2011). However, circulating miR-21 and miR-222 appear highly expressed in individuals with low VO_2max_ when compared to individuals with high VO_2max_ (Bye et al., 2013). These discrepancies may be explained by differences in the exercise protocols used. However, it is becoming apparent that various RNA extraction protocols introduce differences in the expression levels of the measured miRNAs (McAlexander et al., 2013). In addition, hemolysis is a major source of plasma miRNAs (Pritchard et al., 2012) and caution needs to be taken during blood draw and plasma separation to avoid miRNA contamination from red blood cells. The expression of plasma miRNAs derived from hematopoietic cells correlates strongly with hematopoietic cell number (Pritchard et al., 2012). Therefore, changes in circulating miRNA levels within subjects may reflect exercise-induced changes in blood cell numbers (Tanimura et al., 2009; Connes et al., 2013; Tonevitsky et al., 2013) rather than muscle-specific adaptations. Tonevitsky et al. completed a miRNA array in whole blood of trained individuals following a single bout of treadmill exercise. MiR-21-5p, miR-24-2-5p, miR-27a-5p, miR-181a-5p and miR-181b-5p were all regulated immediately post-exercise and during recovery (Tonevitsky et al., 2013). Bioinformatics analysis predicted these miRNAs to target exercise-responsive processes including immune function, apoptosis, membrane trafficking and transcriptional regulation. However, these relationships have not been experimentally validated.

**Table 2:**
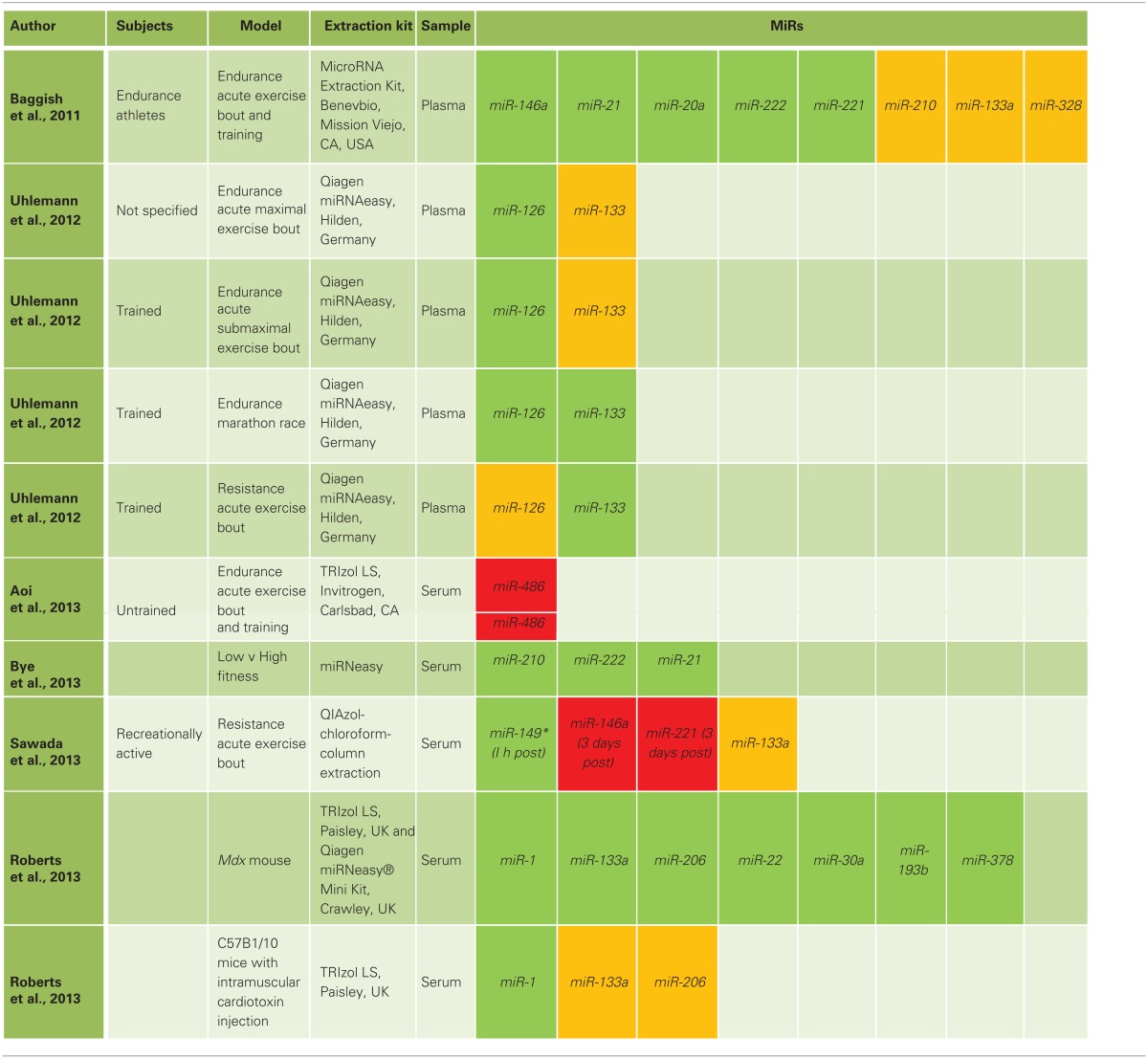
**Regulation of circulating miRNAs by exercise, training, and fitness level**.

Circulating miRNAs regulated by acute exercise were measured immediately post-exercise. Red, downregulated; Green, upregulated; Orange, no change.

An ideal miRNA biomarker candidate for muscle disease or adaptation to exercise should not be expressed by hematopoietic cells but rather be predominantly expressed in the tissue of interest, such as miR-133a (Callis et al., 2008) and miR-210; the latter described as a hypoximiR (Devlin et al., 2011). More work is required to determine whether circulating miRNAs can serve as stable blood-based biomarkers for underlying skeletal muscle diseases and exercise-induced muscle adaptations.

## Concluding remarks

MiRNAs are positive regulators of myogenesis. Their expression levels change following a single bout of exercise and exercise training and following nutritional interventions. A dysregulation of various miRNAs occurs in myopathies, in chronic diseases associated with muscle atrophy as well as with ageing. These observations suggest that skeletal muscle miRNAs play an important role in muscle adaptation and maladaptation. The identification of circulating miRNAs and their regulation following exercise and in disease suggests that they may be useful biomarkers of health and adaptation to treatment interventions. These observations also imply that miRNAs might be amenable to therapeutic intervention. However, at present we have little knowledge relating to how changes in skeletal muscle or circulating miRNAs influence, either directly or indirectly, changes in skeletal muscle regeneration, size, function, metabolism and consequently whole body health. Establishing the causal roles of skeletal muscle miRNAs *in vivo* is now required to significantly advance this exciting field.

### Conflict of interest statement

The authors declare that the research was conducted in the absence of any commercial or financial relationships that could be construed as a potential conflict of interest.

## References

[B1] Alexander M. S. Casar J. C. Motohashi N. Myers J. A. Eisenberg I. Gonzalez R. T. (2011). Regulation of DMD pathology by an ankyrin-encoded miRNA. Skelet. Muscle 1, 27 10.1186/2044-5040-1-27 21824387 PMC3188430

[B2] Alexander M. S. Kawahara G. Motohashi N. Casar J. C. Eisenberg I. Myers J. A. (2013). MicroRNA-199a is induced in dystrophic muscle and affects WNT signaling, cell proliferation, and myogenic differentiation. Cell Death Differ. 20, 1194–1208 10.1038/cdd.2013.62 23764775 PMC3741500

[B3] Allen D. L. Bandstra E. R. Harrison B. C. Thorng S. Stodieck L. S. Kostenuik P. J. (2009). Effects of spaceflight on murine skeletal muscle gene expression. J. Appl. Physiol. 106, 582–595 10.1152/japplphysiol.90780.2008 19074574 PMC2644242

[B4] Aoi W. Ichikawa H. Mune K. Tanimura Y. Mizushima K. Naito Y. (2013). Muscle-enriched microRNA miR-486 decreases in circulation in response to exercise in young men. Front. Physiol. 4:80 10.3389/fphys.2013.00080 23596423 PMC3622901

[B5] Baar K. Esser K. (1999). Phosphorylation of p70^*S6k*^ correlates with increased skeletal muscle mass following resistance exercise. Am. J. Physiol. Cell Physiol. 276, C120–C127 9886927 10.1152/ajpcell.1999.276.1.C120

[B6] Baggish A. L. Hale A. Weiner R. B. Lewis G. D. Systrom D. Wang F. (2011). Dynamic regulation of circulating microRNA during acute exhaustive exercise and sustained aerobic exercise training. J. Physiol. 589, 3983–3994 10.1113/jphysiol.2011.213363 21690193 PMC3179997

[B7] Barres R. Yan J. Egan B. Treebak J. T. Rasmussen M. Fritz T. (2012). Acute exercise remodels promoter methylation in human skeletal muscle. Cell Metab. 15, 405–411 10.1016/j.cmet.2012.01.001 22405075

[B8] Bartel D. P. (2004). MicroRNAs: genomics, biogenesis, mechanism, and function. Cell 116, 281–297 10.1016/S0092-8674(04)00045-5 14744438

[B9] Bassett D. Currie P. D. (2004). Identification of a zebrafish model of muscular dystrophy. Clin. Exp. Pharmacol. Physiol. 31, 537–540 10.1111/j.1440-1681.2004.04030.x 15298547

[B10] Bodine S. C. Latres E. Baumhueter S. Lai V. K. M. Nonez L. Clarke B. A. (2001). Identification of ubiquitin ligases required for skeletal muscle atrophy. Science 294, 1704 10.1126/science.1065874 11679633

[B11] Boehm M. Slack F. (2005). A developmental timing microRNA and its target regulate life span in C. elegans. Science 310, 1954–1957 10.1126/science.1115596 16373574

[B12] Brase J. C. Johannes M. Schlomm T. Fälth M. Haese A. Steuber T. (2011). Circulating miRNAs are correlated with tumor progression in prostate cancer. Int. J. Cancer 128, 608–616 10.1002/ijc.25376 20473869

[B13] Brennecke J. Stark A. Russell R. B. Cohen S. M. (2005). Principles of MicroRNA–target recognition. PLoS Biol. 3:e85 10.1371/journal.pbio.0030085 15723116 PMC1043860

[B14] Bye A. Røsjø H. Aspenes S. T. Condorelli G. Omland T. Wisløff U. (2013). Circulating MicroRNAs and aerobic fitness – The HUNT-Study. PLoS ONE 8:e57496 10.1371/journal.pone.0057496 23469005 PMC3585333

[B15] Cacchiarelli D. Incitti T. Martone J. Cesana M. Cazzella V. Santini T. (2011). miR-31 modulates dystrophin expression: new implications for Duchenne muscular dystrophy therapy. EMBO Rep. 12, 136–141 10.1038/embor.2010.208 21212803 PMC3049433

[B16] Callis T. E. Deng Z. Chen J.-F. Wang D.-Z. (2008). Muscling Through the microRNA World. Exp. Biol. Med. 233, 131–138 10.3181/0709-MR-237 18222968

[B17] Carlson M. E. Suetta C. Conboy M. J. Aagaard P. Mackey A. Kjaer M. (2009). Molecular aging and rejuvenation of human muscle stem cells. EMBO Mol. Med. 1, 381–391 10.1002/emmm.200900045 20049743 PMC2875071

[B18] Cen B. Selvaraj A. Prywes R. (2004). Myocardin/MKL family of SRF coactivators: key regulators of immediate early and muscle specific gene expression. J. Cell. Biochem. 93, 74–82 10.1002/jcb.20199 15352164

[B19] Charvet C. Houbron C. Parlakian A. Giordani J. Lahoute C. Bertrand A. (2006). New role for serum response factor in postnatal skeletal muscle growth and regeneration via the interleukin 4 and insulin-like growth factor 1 pathways. Mol. Cell. Biol. 26, 6664–6674 10.1128/MCB.00138-06 16914747 PMC1592825

[B20] Chen J.-F. Mandel E. M. Thomson J. M. Wu Q. Callis T. E. Hammond S. M. (2006). The role of microRNA-1 and microRNA-133 in skeletal muscle proliferation and differentiation. Nat. Genet. 38, 228–233 10.1038/ng1725 16380711 PMC2538576

[B21] Chen X. Ba Y. Ma L. Cai X. Yin Y. Wang K. (2008). Characterization of microRNAs in serum: a novel class of biomarkers for diagnosis of cancer and other diseases. Cell Res. 18, 997–1006 10.1038/cr.2008.282 18766170

[B22] Colussi C. Mozzetta C. Gurtner A. Illi B. Rosati J. Straino S. (2008). HDAC2 blockade by nitric oxide and histone deacetylase inhibitors reveals a common target in Duchenne muscular dystrophy treatment. Proc. Natl. Acad. Sci. U.S.A. 105, 19183–19187 10.1073/pnas.0805514105 19047631 PMC2614736

[B23] Connes P. Simmonds M. J. Brun J.-F. Baskurt O. K. (2013). Exercise hemorheology: classical data, recent findings and unresolved issues. Clin. Hemorheol. Microcirc. 53, 187–199 23042105 10.3233/CH-2012-1643

[B24] Cuthbertson D. Smith K. Babraj J. Leese G. Waddell T. Atherton P. (2005). Anabolic signaling deficits underlie amino acid resistance of wasting, aging muscle. FASEB J. 19, 422–424 15596483 10.1096/fj.04-2640fje

[B25] D'Alessandra Y. Devanna P. Limana F. Straino S. Di Carlo A. Brambilla P. G. (2010). Circulating microRNAs are new and sensitive biomarkers of myocardial infarction. Eur. Heart J. 31, 2765–2773 10.1093/eurheartj/ehq167 20534597 PMC2980809

[B26] Davidsen P. K. Gallagher I. J. Hartman J. W. Tarnopolsky M. A. Dela F. Helge J. W. (2011). High responders to resistance exercise training demonstrate differential regulation of skeletal muscle microRNA expression. J. Appl. Physiol. 110, 309–317 10.1152/japplphysiol.00901.2010 21030674

[B27] De Lencastre A. Pincus Z. Zhou K. Kato M. Lee S. S. Slack F. J. (2010). MicroRNAs both promote and antagonize longevity in C. elegans. Curr. Biol. 20, 2159–2168 10.1016/j.cub.2010.11.015 21129974 PMC3023310

[B28] Dela F. Ploug T. Handberg A. Petersen L. N. Larsen J. J. Mikines K. J. (1994). Physical training increases muscle GLUT4 protein and mRNA in patients with NIDDM. Diabetes 43, 862–865 10.2337/diabetes.43.7.862 8013748

[B29] Devlin C. Greco S. Martelli F. Ivan M. (2011). miR-210: more than a silent player in hypoxia. IUBMB Life 63, 94–100 21360638 10.1002/iub.427 PMC4497508

[B30] Dong Q. Meng P. Wang T. Qin W. Qin W. Wang F. (2010). MicroRNA Let-7a inhibits proliferation of human prostate cancer cells *in vitro* and *in vivo* by targeting E2F2 and CCND2. PLoS ONE 5:e10147 10.1371/journal.pone.0010147 20418948 PMC2854685

[B31] Drummond M. J. Dreyer H. C. Pennings B. Fry C. S. Dhanani S. Dillon E. L. (2008a). Skeletal muscle protein anabolic response to resistance exercise and essential amino acids is delayed with aging. J. Appl. Physiol. 104, 1452–1461 10.1152/japplphysiol.00021.2008 18323467 PMC2715298

[B32] Drummond M. J. McCarthy J. J. Fry C. S. Esser K. A. Rasmussen B. B. (2008b). Aging differentially affects human skeletal muscle microRNA expression at rest and after an anabolic stimulus of resistance exercise and essential amino acids. Am. J. Physiol. Endocrinol. Metab. 58, E1333–E1340 10.1152/ajpendo.90562.2008 18827171 PMC2603551

[B33] Drummond M. J. McCarthy J. J. Sinha M. Spratt H. M. Volpi E. Esser K. A. (2011). Aging and microRNA expression in human skeletal muscle: a microarray and bioinformatics analysis. Physiol. Genomics 43, 595–603 10.1152/physiolgenomics.00148.2010 20876843 PMC3110890

[B34] Dweep H. Sticht C. Pandey P. Gretz N. (2011). miRWalk – database: prediction of possible miRNA binding sites by “walking” the genes of three genomes. J. Biomed. Inform. 44, 839–847 10.1016/j.jbi.2011.05.002 21605702

[B35] Eisenberg I. Eran A. Nishino I. Moggio M. Lamperti C. Amato A. A. (2007). Distinctive patterns of microRNA expression in primary muscular disorders. Proc. Natl. Acad. Sci. U.S.A. 104, 17016–17021 10.1073/pnas.0708115104 17942673 PMC2040449

[B36] Elia L. Contu R. Quintavalle M. Varrone F. Chimenti C. Russo M. A. (2009). Reciprocal regulation of MicroRNA-1 and insulin-like growth factor-1 signal transduction cascade in cardiac and skeletal muscle in physiological and pathological conditions. Circulation 120, 2377–2385 10.1161/CIRCULATIONAHA.109.879429 19933931 PMC2825656

[B37] Freedman J. Ercan B. Morin K. Liu C.-T. Tamer L. Ayaz L. (2012). The distribution of circulating microRNA and their relation to coronary disease. F1000 Res. [Epub ahead of print]. 10.12688/f1000research.1-50.v1 PMC3752638 24358814

[B38] Fry A. C. (2004). The role of resistance exercise intensity on muscle fibre adaptations. Sports Med. 34, 663–679 10.2165/00007256-200434100-00004 15335243

[B39] Gambardella S. Rinaldi F. Lepore S. M. Viola A. Loro E. Angelini C. (2010). Overexpression of microRNA-206 in the skeletal muscle from myotonic dystrophy type 1 patients. J. Transl. Med. 8, 48 10.1186/1479-5876-8-48 20487562 PMC2880982

[B40] Gilad S. Meiri E. Yogev Y. Benjamin S. Lebanony D. Yerushalmi N. (2008). Serum MicroRNAs are promising novel biomarkers. PLoS ONE 3:e3148 10.1371/journal.pone.0003148 18773077 PMC2519789

[B41] Greco S. Perfetti A. Fasanaro P. Cardani R. Capogrossi M. C. Meola G. (2012). Deregulated microRNAs in myotonic dystrophy type 2. PLoS ONE 7:e39732 10.1371/journal.pone.0039732 22768114 PMC3387258

[B42] Greco S. Simone M. D. Colussi C. Zaccagnini G. Fasanaro P. Pescatori M. (2009). Common micro-RNA signature in skeletal muscle damage and regeneration induced by Duchenne muscular dystrophy and acute ischemia. FASEB J. 23, 3335–3346 10.1096/fj.08-128579 19528256

[B43] Hamrick M. W. Herberg S. Arounleut P. He H.-Z. Shiver A. Qi R.-Q. (2010). The adipokine leptin increases skeletal muscle mass and significantly alters skeletal muscle miRNA expression profile in aged mice. Biochem. Biophys. Res. Commun. 400, 379–383 10.1016/j.bbrc.2010.08.079 20800581 PMC3740337

[B44] Hasten D. L. Pak-Loduca J. Obert K. A. Yarasheski K. E. (2000). Resistance exercise acutely increases MHC and mixed muscle protein synthesis rates in 78–84 and 23–32 yr olds. Am. J. Physiol. Endocrinol. Metab. 278, E620–E626 10751194 10.1152/ajpendo.2000.278.4.E620

[B45] Hawke T. J. (2005). Muscle stem cells and exercise training. Exerc. Sport Sci. Rev. 33, 63–68 10.1097/00003677-200504000-00002 15821426

[B46] Hawley J. A. Hargreaves M. Zierath J. R. (2006). Signalling mechanisms in skeletal muscle: role in substrate selection and muscle adaptation. Essays Biochem. 42, 1–12 10.1042/bse0420001 17144876

[B47] Hu Z. Bruno A. E. (2011). The Influence of 3′UTRs on MicroRNA function inferred from human SNP data. Comp. Funct. Genomics 2011:910769 10.1155/2011/910769 22110399 PMC3202110

[B48] Huang Z. Huang D. Ni S. Peng Z. Sheng W. Du X. (2010). Plasma microRNAs are promising novel biomarkers for early detection of colorectal cancer. Int. J. Cancer 127, 118–126 10.1002/ijc.25007 19876917

[B49] Huili G. Ingolia N. T. Weissman J. S. Bartel D. P. (2010). Mammalian microRNAs predominantly act to decrease target mRNA levels. Nature 466, 835–840 10.1038/nature09267 20703300 PMC2990499

[B50] Humphreys D. T. Westman B. J. Martin D. I. K. Preiss T. (2005). MicroRNAs control translation initiation by inhibiting eukaryotic initiation factor 4E/cap and poly(A) tail function. (English). Proc. Natl. Acad. Sci. U.S.A. 102, 16961–16966 10.1073/pnas.0506482102 16287976 PMC1287990

[B51] Ibáñez-Ventoso C. Yang M. Guo S. Robins H. Padgett R. W. Driscoll M. (2006). Modulated microRNA expression during adult lifespan in *Caenorhabditis elegans*. Aging Cell 5, 235–246 10.1111/j.1474-9726.2006.00210.x 16842496

[B52] Johnson C. D. Esquela-Kerscher A. Stefani G. Byrom M. Kelnar K. Ovcharenko D. (2007). The let-7 microRNA represses cell proliferation pathways in human cells. Cancer Res. 67, 7713–7722 10.1158/0008-5472.CAN-07-1083 17699775

[B53] Katsanos C. S. Chinkes D. L. Paddon-Jones D. Zhang X.-J. Aarsland A. Wolfe R. R. (2008). Whey protein ingestion in elderly persons results in greater muscle protein accrual than ingestion of its constituent essential amino acid content. Nutr. Res. 28, 651–658 10.1016/j.nutres.2008.06.007 19083472 PMC2612691

[B54] Keller C. Steensberg A. Pilegaard H. Osada T. Saltin B. Pedersen B. K. (2001). Transcriptional activation of the IL-6 gene in human contracting skeletal muscle: influence of muscle glycogen content. FASEB J. 15, 2748–2750 11687509 10.1096/fj.01-0507fje

[B55] Keller P. Vollaard N. B. Gustafsson T. Gallagher I. J. Sundberg C. J. Rankinen T. (2011). A transcriptional map of the impact of endurance exercise training on skeletal muscle phenotype. J. Appl. Physiol. 110, 46–59 10.1152/japplphysiol.00634.2010 20930125 PMC3253010

[B56] Khvorova A. Reynolds A. Jayasena S. D. (2003). Functional siRNAs and miRNAs exhibit strand bias. Cell 115, 209 10.1016/S0092-8674(03)00801-8 14567918

[B57] Kim E. Cook-Mills J. Morgan G. Sredni S. T. Pachman L. M. (2012). Increased expression of vascular cell adhesion molecule 1 in muscle biopsy samples from juvenile dermatomyositis patients with short duration of untreated disease is regulated by miR-126. Arthritis Rheum. 64, 3809–3817 10.1002/art.34606 22740338 PMC3469762

[B58] Kim H. K. Lee Y. S. Sivaprasad U. Malhotra A. Dutta A. (2006). Muscle-specific microRNA miR-206 promotes muscle differentiation. J. Cell Biol. 174, 677–687 10.1083/jcb.200603008 16923828 PMC2064311

[B59] Koopman R. Gleeson B. Gijsen A. P. Groen B. Senden J. M. G. Rennie M. J. (2011). Post-exercise protein synthesis rates are only marginally higher in type I compared with type II muscle fibres following resistance-type exercise. Eur. J. Appl. Physiol. 111, 1871–1878 10.1007/s00421-010-1808-9 21234594 PMC3156941

[B59a] Kosaka N. Iguchi H. Ochiya T. (2010). Circulating microRNA in body fluid: a new potential biomarker for cancer diagnosis and prognosis. Cancer Sci. 101, 2087–2092 10.1111/j.1349-7006.2010.01650.x 20624164 PMC11159200

[B60] Kramer H. F. Goodyear L. J. (2007). Exercise, MAPK, and NF-kappaB signaling in skeletal muscle. J. Appl. Physiol. 103, 388–395 10.1152/japplphysiol.00085.2007 17303713

[B61] Kumar V. Selby A. Rankin D. Patel R. Atherton P. Hildebrandt W. (2009). Age-related differences in the dose-response relationship of muscle protein synthesis to resistance exercise in young and old men. J. Physiol. 587, 211–217 10.1113/jphysiol.2008.164483 19001042 PMC2670034

[B62] Lee I. Ajay S. S. Yook J. I. Kim H. S. Hong S. H. Kim N. H. (2009). New class of microRNA targets containing simultaneous 5'-UTR and 3'-UTR interaction sites. Genome Res. 19, 1175–1183 10.1101/gr.089367.108 19336450 PMC2704433

[B63] Lee R. C. Feinbaum R. L. Ambros V. (1993). The C. elegans heterochronic gene lin-4 encodes small RNAs with antisense complementarity to lin-14. Cell 75, 843–854 10.1016/0092-8674(93)90529-Y 8252621

[B64] Lee S.-T. Chu K. Oh H.-J. Im W.-S. Lim J.-Y. Kim S.-K. (2011). Let-7 microRNA inhibits the proliferation of human glioblastoma cells. J. Neurooncol. 102, 19–24 10.1007/s11060-010-0286-6 20607356

[B65] Lee S. W. Dai G. Hu Z. Wang X. Du J. Mitch W. E. (2004). Regulation of muscle protein degradation: coordinated control of apoptotic and Ubiquitin-Proteasome systems by Phosphatidylinositol 3 kinase. J. Am. Soc. Nephrol. 15, 1537–1545 10.1097/01.ASN.0000127211.86206.E1 15153564

[B66] Lee Y. Ahn C. Han J. Choi H. Kim J. Yim J. (2003). The nuclear RNase III drosha initiates microRNA processing. Nature 425, 415–419 10.1038/nature01957 14508493

[B67] Léger B. Cartoni R. Praz M. Lamon S. Dériaz O. Crettenand A. (2006). Akt signalling through GSK-3beta, mTOR and Foxo1 is involved in human skeletal muscle hypertrophy and atrophy. J. Physiol. 576, 923–933 10.1113/jphysiol.2006.116715 16916907 PMC1890416

[B68] Le Grand F. Jones A. E. Seale V. Scimè A. Rudnicki M. A. (2009). Wnt7a activates the planar cell polarity pathway to drive the symmetric expansion of satellite stem cells. Cell Stem Cell 4, 535–547 10.1016/j.stem.2009.03.013 19497282 PMC2743383

[B69] Lewis A. Riddoch-Contreras J. Natanek S. A. Donaldson A. Man W. D. Moxham J. (2012). Downregulation of the serum response factor/miR-1 axis in the quadriceps of patients with COPD. Thorax 67, 26–34 10.1136/thoraxjnl-2011-200309 21998125 PMC3240776

[B70] Lewis B. P. Burge C. B. Bartel D. P. (2005). Conserved seed pairing, often flanked by adenosines, indicates that thousands of human genes are MicroRNA targets. Cell 120, 15–20 10.1016/j.cell.2004.12.035 15652477

[B71] Liu N. Williams A. H. Maxeiner J. M. Bezprozvannaya S. Shelton J. M. Richardson J. A. (2012). microRNA-206 promotes skeletal muscle regeneration and delays progression of Duchenne muscular dystrophy in mice. J. Clin. Invest. 122, 2054–2065 10.1172/JCI62656 22546853 PMC3366415

[B72] Lund E. Güttinger S. Calado A. Dahlberg J. E. Kutay U. (2004). Nuclear export of MicroRNA precursors. Science 303, 95–98 10.1126/science.1090599 14631048

[B73] McAlexander M. A. Phillips M. J. Witwer K. W. (2013). Comparison of methods for miRNA extraction from plasma and quantitative recovery of RNA from plasma and cerebrospinal fluid. Front. Genet. 4:83 10.3389/fgene.2013.00083 23720669 PMC3655275

[B74] McCarthy J. J. Esser K. A. (2007). MicroRNA-1 and microRNA-133a expression are decreased during skeletal muscle hypertrophy. J. Appl. Physiol. 102, 306–313 10.1152/japplphysiol.00932.2006 17008435

[B75] McCarthy J. J. Esser K. A. Andrade F. H. (2007). MicroRNA-206 is overexpressed in the diaphragm but not the hindlimb muscle of mdx mouse. Am. J. Physiol. Cell Physiol. 62, C451–C457 10.1152/ajpcell.00077.2007 17459947

[B76] McCarthy J. J. Esser K. A. Peterson C. A. Dupont-Versteegden E. E. (2009). Evidence of MyomiR network regulation of β-myosin heavy chain gene expression during skeletal muscle atrophy. Physiol. Genomics 39, 219–226 10.1152/physiolgenomics.00042.2009 19690046 PMC2789671

[B77] McGee S. L. Fairlie E. Garnham A. P. Hargreaves M. (2009). Exercise-induced histone modifications in human skeletal muscle. J. Physiol. 587, 5951–5958 10.1113/jphysiol.2009.181065 19884317 PMC2808551

[B78] McGee S. L. Sparling D. Olson A. L. Hargreaves M. (2006). Exercise increases MEF2- and GEF DNA-binding activity in human skeletal muscle. FASEB J. 20, 348–349 16368714 10.1096/fj.05-4671fje

[B79] Menzies F. M. Ince P. G. Shaw P. J. (2002). Mitochondrial involvement in amyotrophic lateral sclerosis. Neurochem. Int. 40, 543–551 10.1016/S0197-0186(01)00125-5 11850111

[B80] Miano J. M. Long X. Fujiwara K. (2007). Serum response factor: master regulator of the actin cytoskeleton and contractile apparatus. Am. J. Physiol. Cell Physiol. 292, C70–C81 10.1152/ajpcell.00386.2006 16928770

[B81] Mitchell P. S. Parkin R. K. Kroh E. M. Fritz B. R. Wyman S. K. Pogosova-Agadjanyan E. L. (2008). Circulating microRNAs as stable blood-based markers for cancer detection. Proc. Natl. Acad. Sci. U.S.A. 105, 10513–10518 10.1073/pnas.0804549105 18663219 PMC2492472

[B82] Nakajima K. Takeoka M. Mori M. Hashimoto S. Sakurai A. Nose H. (2010). Exercise effects on methylation of ASC gene. Int. J. Sports Med. 31, 671–675 10.1055/s-0029-1246140 20200803

[B83] Nakasa T. Ishikawa M. Shi M. Shibuya H. Adachi N. Ochi M. (2010). Acceleration of muscle regeneration by local injection of muscle-specific microRNAs in rat skeletal muscle injury model. J. Cell. Mol. Med. 14, 2495–2505 10.1111/j.1582-4934.2009.00898.x 19754672 PMC3823166

[B84] Nielsen S. Scheele C. Yfanti C. Akerstrom T. Nielsen A. R. Pedersen B. K. (2010). Muscle specific microRNAs are regulated by endurance exercise in human skeletal muscle. J. Physiol. 588, 4029–4037 10.1113/jphysiol.2010.189860 20724368 PMC3000590

[B85] Nishino J. Kim I. Chada K. Morrison S. J. (2008). Hmga2 promotes neural stem cell self-renewal in young but not old mice by reducing p16Ink4a and p19Arf expression. Cell 135, 227–239 10.1016/j.cell.2008.09.017 18957199 PMC2582221

[B86] Olsen P. H. Ambros V. (1999). The lin-4 regulatory RNA controls developmental timing in caenorhabditis elegans by blocking LIN-14 protein synthesis after the initiation of translation. Dev. Biol. 216, 671–680 10.1006/dbio.1999.9523 10642801

[B87] Pasinelli P. Brown R. H. (2006). Molecular biology of amyotrophic lateral sclerosis: insights from genetics. Nat. Rev. Neurosci. 7, 710–723 10.1038/nrn1971 16924260

[B88] Perbellini R. Greco S. Sarra-Ferraris G. Cardani R. Capogrossi M. C. Meola G. (2011). Dysregulation and cellular mislocalization of specific miRNAs in myotonic dystrophy type 1. Neuromuscul. Disord. 21, 81–88 10.1016/j.nmd.2010.11.012 21169019

[B89] Phillips S. M. (2009). Physiologic and molecular bases of muscle hypertrophy and atrophy: impact of resistance exercise on human skeletal muscle (protein and exercise dose effects). Appl. Physiol. Nutr. Metab. 34, 403–410 10.1139/H09-042 19448706

[B90] Pigati L. Yaddanapudi S. C. S. Iyengar R. Dong-Ja K. Hearn S. A. Danforth D. (2010). Selective release of MicroRNA species from normal and malignant mammary epithelial cells. PLoS ONE 5:e13515 10.1371/journal.pone.0013515 20976003 PMC2958125

[B91] Pillai R. S. Bhattacharyya S. N. Artus C. G. Zoller T. Cougot N. Basyuk E. (2005). Inhibition of translational initiation by Let-7 MicroRNA in human cells. Science 309, 1573–1576 10.1126/science.1115079 16081698

[B92] Polesskaya A. Seale P. Rudnicki M. A. (2003). Wnt signaling induces the myogenic specification of resident CD45+ adult stem cells during muscle regeneration. Cell 113, 841–852 10.1016/S0092-8674(03)00437-9 12837243

[B93] Pritchard C. C. Kroh E. Wood B. Arroyo J. D. Dougherty K. J. Miyaji M. M. (2012). Blood cell origin of circulating micrornas: a cautionary note for cancer biomarker studies. Cancer Prev. Res. 5, 492–497 10.1158/1940-6207.CAPR-11-0370 22158052 PMC4186243

[B94] Rao P. K. Kumar R. M. Farkhondeh M. Baskerville S. Lodish H. F. (2006). Myogenic factors that regulate expression of muscle-specific microRNAs. Proc. Natl. Acad. Sci. U.S.A. 103, 8721–8726 10.1073/pnas.0602831103 16731620 PMC1482645

[B95] Reinhart B. J. Slack F. J. Basson M. Pasquinelli A. E. Bettinger J. C. Rougvie A. E. (2000). The 21-nucleotide let-7 RNA regulates developmental timing in *Caenorhabditis Elegans*. Nature 403, 901–906 10.1038/35002607 10706289

[B96] Roberts T. C. Godfrey C. McClorey G. Vader P. Briggs D. Gardiner C. (2013). Extracellular microRNAs are dynamic non-vesicular biomarkers of muscle turnover. Nucleic Acids Res. [Epub ahead of print]. 10.1093/nar/gkt724 23945935 PMC3814379

[B97] Rosenberg M. I. Georges S. A. Asawachaicharn A. Analau E. Tapscott S. J. (2006). MyoD inhibits Fstl1 and Utrn expression by inducing transcription of miR-206. J. Cell Biol. 175, 77–85 10.1083/jcb.200603039 17030984 PMC2064500

[B98] Rudnicki M. A. Jaenisch R. (1995). The MyoD family of transcription factors and skeletal myogenesis. Bioessays 17, 203–209 10.1002/bies.950170306 7748174

[B99] Russell A. P. (2010). Molecular regulation of skeletal muscle mass. Clin. Exp. Pharmacol. Physiol. 37, 378–384 10.1111/j.1440-1681.2009.05265.x 19650790

[B100] Russell A. P. Feilchenfeldt J. Schreiber S. Praz M. Crettenand A. Gobelet C. (2003). Endurance training in humans leads to fiber type-specific increases in levels of peroxisome proliferator-activated receptor-gamma coactivator-1 and peroxisome proliferator-activated receptor-alpha in skeletal muscle. Diabetes 52, 2874–2881 10.2337/diabetes.52.12.2874 14633846

[B101] Russell A. P. Hesselink M. K. Lo S. K. Schrauwen P. (2005). Regulation of metabolic transcriptional co-activators and transcription factors with acute exercise. FASEB J. 19, 986–988 10.1096/fj.04-3168fje 15814608

[B102] Russell A. P. Lamon S. Boon H. Wada S. Guller I. Brown E. L. (2013). Regulation of miRNAs in human skeletal muscle following acute endurance exercise and short term endurance training. J. Physiol. 591, 4637–4653 10.1113/jphysiol.2013.255695 23798494 PMC3784204

[B103] Russell A. P. Wada S. Vergani L. Hock M. B. Lamon S. Leger B. (2012). Disruption of skeletal muscle mitochondrial network genes and miRNAs in amyotrophic lateral sclerosis. Neurobiol. Dis. 49C, 107–117 22975021 10.1016/j.nbd.2012.08.015

[B104] Sabourin L. A. Rudnicki M. A. (2000). The molecular regulation of myogenesis. Clin. Genet. 57, 16–25 10.1034/j.1399-0004.2000.570103.x 10733231

[B105] Sawada S. Kon M. Wada S. Ushida T. Suzuki K. Akimoto T. (2013). Profiling of Circulating MicroRNAs after a bout of acute resistance exercise in humans. PLoS ONE 8:e70823 10.1371/journal.pone.0070823 23923026 PMC3726615

[B106] Schabort E. J. Van Der Merwe M. Loos B. Moore F. P. Niesler C. U. (2009). TGF-beta's delay skeletal muscle progenitor cell differentiation in an isoform-independent manner. Exp. Cell Res. 315, 373–384 10.1016/j.yexcr.2008.10.037 19038250

[B107] Short K. R. Vittone J. L. Bigelow M. L. Proctor D. N. Rizza R. A. Coenen-Schimke J. M. (2003). Impact of aerobic exercise training on age-related changes in insulin sensitivity and muscle oxidative capacity. Diabetes 52, 1888–1896 10.2337/diabetes.52.8.1888 12882902

[B108] Small E. M. O'Rourke J. R. Moresi V. Sutherland L. B. McAnally J. Gerard R. D. (2010). Regulation of PI3-kinase/Akt signaling by muscle-enriched microRNA-486. Proc. Natl. Acad. Sci. U.S.A. 107, 4218–4223 10.1073/pnas.1000300107 20142475 PMC2840099

[B109] Sood P. Krek A. Zavolan M. Macino G. Rajewsky N. (2006). Cell-type-specific signatures of microRNAs on target mRNA expression. Proc. Natl. Acad. Sci. U.S.A. 103, 2746–2751 10.1073/pnas.0511045103 16477010 PMC1413820

[B110] Sweetman D. Goljanek K. Rathjen T. Oustanina S. Braun T. Dalmay T. (2008). Specific requirements of MRFs for the expression of muscle specific microRNAs, miR-1, miR-206 and miR-133. Dev. Biol. 321, 491–499 10.1016/j.ydbio.2008.06.019 18619954

[B111] Sylvius N. Bonne G. Straatman K. Reddy T. Gant T. W. Shackleton S. (2011). MicroRNA expression profiling in patients with lamin A/C-associated muscular dystrophy. FASEB J. 25, 3966–3978 10.1096/fj.11-182915 21840938

[B112] Symons T. B. Sheffield-Moore M. Wolfe R. R. Paddon-Jones D. (2009). A moderate serving of high-quality protein maximally stimulates skeletal muscle protein synthesis in young and elderly subjects. J. Am. Diet. Assoc. 109, 1582–1586 10.1016/j.jada.2009.06.369 19699838 PMC3197704

[B113] Tajbakhsh S. Bober E. Babinet C. Pournin S. Arnold H. Buckingham M. (1996). Gene targeting the myf-5 locus with nlacZ reveals expression of this myogenic factor in mature skeletal muscle fibres as well as early embryonic muscle. Dev. Dyn. 206, 291–300 10.1002/(SICI)1097-0177(199607)206:3<291::AID-AJA6>3.0.CO;2-D 8896984

[B114] Tanimura Y. Kon M. Shimizu K. Kimura F. Kono I. Ajisaka R. (2009). Effect of 6-day intense Kendo training on lymphocyte counts and its expression of CD95. Eur. J. Appl. Physiol. 107, 227–233 10.1007/s00421-009-1119-1 19568765

[B115] Terzis G. Georgiadis G. Stratakos G. Vogiatzis I. Kavouras S. Manta P. (2008). Resistance exercise-induced increase in muscle mass correlates with p70S6 kinase phosphorylation in human subjects. Eur. J. Appl. Physiol. 102, 145–152 10.1007/s00421-007-0564-y 17874120

[B116] Timmons J. A. Knudsen S. Rankinen T. Koch L. G. Sarzynski M. Jensen T. (2010). Using molecular classification to predict gains in maximal aerobic capacity following endurance exercise training in humans. J. Appl. Physiol. 108, 1487–1496 10.1152/japplphysiol.01295.2009 20133430 PMC2886694

[B117] Tintignac L. A. Lagirand J. Batonnet S. Sirri V. Leibovitch M. P. Leibovitch S. A. (2005). Degradation of MyoD mediated by the SCF (MAFbx) ubiquitin ligase. J. Biol. Chem. 280, 2847–2856 10.1074/jbc.M411346200 15531760

[B118] Tonevitsky A. Maltseva D. Abbasi A. Samatov T. Sakharov D. Shkurnikov M. (2013). Dynamically regulated miRNA-mRNA networks revealed by exercise. BMC Physiol. 13:9 10.1186/1472-6793-13-9 PMC3681679 24219008

[B119] Turchinovich A. Weiz L. Langheinz A. Burwinkel B. (2011). Characterization of extracellular circulating microRNA. Nucleic Acids Res. 39, 7223–7233 10.1093/nar/gkr254 21609964 PMC3167594

[B120] Uhlemann M. Möbius-Winkler S. Fikenzer S. Adam J. Redlich M. Möhlenkamp S. (2012). Circulating microRNA-126 increases after different forms of endurance exercise in healthy adults. Eur. J. Prev. Cardiol. [Epub ahead of print]. 10.1177/2047487312467902 23150891

[B121] Valadi H. Ekstrom K. Bossios A. Sjostrand M. Lee J. J. Lotvall J. O. (2007). Exosome-mediated transfer of mRNAs and microRNAs is a novel mechanism of genetic exchange between cells. Nat. Cell Biol. 9, 654–659 10.1038/ncb1596 17486113

[B122] Vickers K. C. Palmisano B. T. Shoucri B. M. Shamburek R. D. Remaley A. T. (2011). MicroRNAs are transported in plasma and delivered to recipient cells by high-density lipoproteins. Nat. Cell Biol. 13, 423–433 10.1038/ncb2210 21423178 PMC3074610

[B123] Wadley G. D. Konstantopoulos N. Macaulay L. Howlett K. F. Garnham A. Hargreaves M. (2007). Increased insulin-stimulated Akt pSer473 and cytosolic SHP2 protein abundance in human skeletal muscle following acute exercise and short-term training. J. Appl. Physiol. 102, 1624–1631 10.1152/japplphysiol.00821.2006 17185494

[B124] Wightman B. Ha I. Ruvkun G. (2004). Posttranscriptional regulation of the heterochronic gene lin-14 by lin-4 mediates temporal pattern formation in C. elegans. Cell 116, 855–862 10.1016/0092-8674(93)90530-4 8252622

[B125] Yamakuchi M. Lowenstein C. J. (2009). MiR-34, SIRT1, and p53: the feedback loop. Cell Cycle 8, 712–715 10.4161/cc.8.5.7753 19221490

[B126] Yarasheski K. E. Zachwieja J. J. Bier D. M. (1993). Acute effects of resistance exercise on muscle protein synthesis rate in young and elderly men and women. Am. J. Physiol. Endocrinol. Metab. 265, E210–E214 8368290 10.1152/ajpendo.1993.265.2.E210

[B127] Yuasa K. Hagiwara Y. Ando M. Nakamura A. Takeda S. Hijikata T. (2008). MicroRNA-206 is highly expressed in newly formed muscle fibers: implications regarding potential for muscle regeneration and maturation in muscular dystrophy. Cell Struct. Funct. 33, 163–169 10.1247/csf.08022 18827405

[B128] Zhang Y. Liu D. Chen X. Li J. Li L. Bian Z. (2010). Secreted monocytic miR-150 enhances targeted endothelial cell migration. Mol. Cell 39, 133–144 10.1016/j.molcel.2010.06.010 20603081

[B129] Zhao C. Sun G. Li S. Lang M.-F. Yang S. Li W. (2010). MicroRNA let-7b regulates neural stem cell proliferation and differentiation by targeting nuclear receptor TLX signaling. Proc. Natl. Acad. Sci. U.S.A. 107, 1876–1881 10.1073/pnas.0908750107 20133835 PMC2836616

